# Respiratory muscles: myths and secrets

**DOI:** 10.1590/S1806-37132015000200002

**Published:** 2015

**Authors:** Eloara Vieira Machado Ferreira

**Affiliations:** 1 Pulmonologist. Pulmonary Circulation Group/Pulmonary Function and Exercise Physiology Section, Department of Pulmonology, Universidade Federal de São Paulo/Escola Paulista de Medicina - UNIFESP/EPM, Federal University of São Paulo/Paulista School of Medicine - São Paulo, Brazil

Why is it so important that pulmonologists are familiar with respiratory muscle assessment? 

Respiratory muscle weakness is associated with increased respiratory workload and reduced or interrupted (central or peripheral) neural stimulation. In healthy individuals (in whom central respiratory drive is normal), the respiratory muscle strength required in order to drive the respiratory system must be greater than the sum of the work imposed by the lungs, rib cage, and airways.^(^ 1 ^)^ An imbalance between the respiratory workload and respiratory muscle strength results in progressive respiratory muscle weakness, which can progress to alveolar hypoventilation and respiratory failure depending on its severity. In most cases, the inspiratory muscles (the most important of which is the diaphragm) are affected first, because of their active role in breathing.^(^ 2 ^)^

Several diseases, particularly neuromuscular diseases, can affect the respiratory muscles. However, systemic inflammation (autoimmune rheumatic diseases), heart failure, and pulmonary impairment, observed in obstructive diseases with lung hyperinflation, in restrictive diseases, and in cases of rib cage deformities, can also adversely affect the respiratory muscles.^(^ 3 ^)^ Therefore, respiratory muscle assessment can be one of the steps in the investigation of dyspnea of unknown cause or in the investigation of the clinical and functional dissociation in patients with chronic respiratory failure. 

In the initial evaluation of respiratory muscle strength, easily applied and widely available methods should be prioritized, overall evaluation of respiratory muscles being given priority over a more specific respiratory muscle assessment. Therefore, MIP and MEP measurements play a central role in the diagnostic evaluation of respiratory muscle strength. Values of MIP and MEP within the normal range rule out respiratory muscle weakness. However, low MIP and MEP values do not unequivocally confirm the presence of respiratory muscle weakness, because they might be related to technical problems or underexertion; therefore, further investigation is required in order to confirm the diagnosis. Steier et al.^(^ 4 ^)^ showed that the use of MIP and MEP alone in the evaluation of patients with neuromuscular disease or patients with dyspnea of unknown cause can lead to overdiagnosis of respiratory muscle weakness, whereas a combination of methods reduces false-positive results by 30%. 

In the current issue of the Brazilian Journal of Pulmonology, Caruso et al.^(^ 5 ^)^ present the various methods of respiratory muscle assessment. The division of the methods into volitional and nonvolitional respiratory muscle tests and their progressive sequencing (ranging from simple, noninvasive tests to tests that are more complex) facilitate the understanding of the tests and aid in choosing the test that is most suitable for the suspected diagnosis. It is of note that the authors addressed the increased use of diaphragmatic ultrasound in determining inspiratory muscle weakness. The advantage of ultrasound is that it requires equipment that is widely available, although it requires an operator who is familiar with the technique. Ultrasound can be used in order to evaluate diaphragmatic structure and function and can be performed in an outpatient or hospital setting.^(^ 5 ^)^ However, certain diseases require methods that are more complex for an accurate diagnosis, including electrical or magnetic phrenic nerve stimulation and (surface or needle) electromyography, the latter being able to evaluate the diaphragm and different inspiratory and expiratory muscles separately.^(^ 2 ^)^

It should be noted that respiratory muscle assessment can be performed in the pediatric population. However, volitional respiratory muscle testing is not feasible in infancy and early childhood in particular. Therefore, invasive techniques such as sniff transdiaphragmatic pressure during crying spells and sniff nasal inspiratory pressure are required and can be performed in children over 4 years of age.^(^ 6 ^)^

Despite being outside the scope of the study by Caruso et al.,^(^ 5 ^)^ tests such as pulmonary function testing, arterial blood gas analysis, nocturnal oximetry, and polysomnography are important in the initial investigation and monitoring of disease progression, as well as in informing decisions regarding noninvasive ventilation.^(^ 7 ^,^ 8 ^)^ A diagnosis of inspiratory muscle weakness is unlikely in patients who present with preserved VC and are suspected of having the condition. In such patients, inspiratory capacity is reduced, resulting in reduced TLC with virtually unchanged functional residual capacity.^(^ 9 ^)^ The onset of nocturnal hypoventilation with hypoxemia and the presence of hypercapnia indicate disease severity and a risk of respiratory failure.^(^ 9 ^,^ 10 ^)^

Regarding the answer to the initial question, it is essential that pulmonologists understand the pathophysiological mechanisms involved in respiratory muscle impairment; that they are familiar with the wide range of differential diagnoses (especially when investigating dyspnea); and that they are able to intervene when serial evaluations reveal signs of complications. In addition, it is crucial that pulmonologists do not take a simplistic approach to respiratory muscle assessment by requesting MIP and MEP measurements to determine the presence or absence of respiratory muscle weakness. Therefore, on the basis of the various methods presented by Caruso et al.,^(^ 5 ^)^ I herein propose a diagnostic algorithm for respiratory muscle weakness (Figure 1). 

**Figure 1 -:**
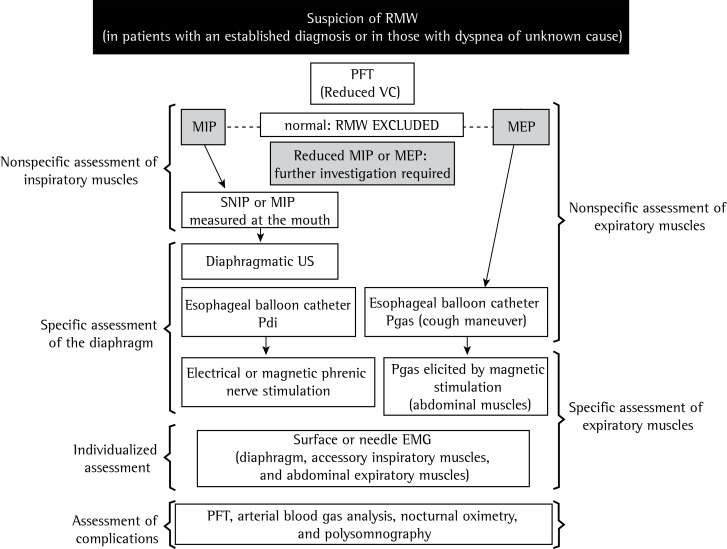
Diagnostic algorithm for respiratory muscle weakness. RMW: respiratory muscle weakness; PFT: pulmonary function testing; SNIP: sniff nasal inspiratory pressure; US: ultrasound; Pdi: transdiaphragmatic pressure; Pgas: gastric pressure; and EMG: e

## References

[1] Fauroux B, Khirani S (2014). Neuromuscular disease and respiratory physiology in children: putting lung function into perspective. Respirology.

[2] Ratnovsky A, Elad D, Halpern P (2008). Mechanics of respiratory muscles. Respir Physiol Neurobiol.

[3] Laghi F, Tobin MJ (2003). Disorders of the respiratory muscles. Am J Respir Crit Care Med.

[4] Caruso P, Albuquerque AL, Santana PV, Cardenas LZ, Ferreira JG, Prina E (2015). Diagnostic methods to assess inspiratory and expiratory muscle strength. J Bras Pneumol.

[5] Steier J, Kaul S, Seymour J, Jolley C, Rafferty G, Man W (2007). The value of multiple tests of respiratory muscle strength. Thorax.

[6] Sarwal A, Walker FO, Cartwright MS (2013). Neuromuscular ultrasound for evaluation of the diaphragm. Muscle Nerve.

[7] Marques TB, Neves Jde C, Portes LA, Salge JM, Zanoteli E, Reed UC (2014). Air stacking: effects on pulmonary function in patients with spinal muscular atrophy and in patients with congenital muscular dystrophy. J Bras Pneumol.

[8] Lira CA, Minozzo FC, Sousa BS, Vancini RL, Andrade Mdos S, Quadros AA (2013). Lung function in post-poliomyelitis syndrome: a cross-sectional study. J Bras Pneumol.

[9] Flaminiano LE, Celli BR (2001). Respiratory muscle testing. Clin Chest Med.

[10] Fauroux B (2003). Respiratory muscle testing in children. Paediatr Respir Rev.

